# Pulmonary magnetic resonance-guided online adaptive radiotherapy of locally advanced non-small-cell lung cancer: the PUMA trial

**DOI:** 10.1186/s13014-023-02258-9

**Published:** 2023-05-04

**Authors:** Sebastian Regnery, Chiara de Colle, Chukwuka Eze, Stefanie Corradini, Christian Thieke, Oliver Sedlaczek, Heinz-Peter Schlemmer, Julien Dinkel, Ferdinand Seith, Annette Kopp-Schneider, Clarissa Gillmann, C. Katharina Renkamp, Guillaume Landry, Daniela Thorwarth, Daniel Zips, Claus Belka, Oliver Jäkel, Jürgen Debus, Juliane Hörner-Rieber

**Affiliations:** 1 Department of Radiation Oncology, Heidelberg University Hospital, Im Neuenheimer Feld 400, 69120 Heidelberg, Germany; 2 National Center for Radiation Oncology (NCRO), Heidelberg Institute for Radiation Oncology (HIRO), Im Neuenheimer Feld 400, 69120 Heidelberg, Germany; 3 Department of Radiation Oncology, Heidelberg Ion-Beam Therapy Center (HIT), Heidelberg University Hospital, Heidelberg, Germany; 4 National Center for Tumor diseases (NCT), Heidelberg, Germany; 5 Clinical Cooperation Unit Radiation Oncology, German Cancer Research Center (DKFZ), Heidelberg, Germany; 6 Department of Radiation Oncology, University Hospital Tübingen, Tübingen, Germany; 7 Department of Radiation Oncology, University Hospital, LMU Munich, Munich, Germany; 8 Division of Radiology, German Cancer Research Center (DKFZ), Heidelberg, Germany; 9 Department of Radiology, LMU Munich, Munich, Germany; 10 Department of Radiology, University Hospital Tübingen, Tübingen, Germany; 11 Division of Biostatistics, German Cancer Research Center (DKFZ), Heidelberg, Germany; 12 Division of Medical Physics in Radiation Oncology, German Cancer Research Center (DKFZ), Heidelberg, Germany; 13 Section for Biomedical Physics, Department of Radiation Oncology, University Hospital Tübingen, Tübingen, Germany

**Keywords:** Non-small-cell lung carcinoma, Lung Cancer, Image-guided Radiotherapy, MRI, MR-guided Radiotherapy, Adaptation, Gating, Feasibility study

## Abstract

**Background:**

Patients with locally-advanced non-small-cell lung cancer (LA-NSCLC) are often ineligible for surgery, so that definitive chemoradiotherapy (CRT) represents the treatment of choice. Nevertheless, long-term tumor control is often not achieved. Intensification of radiotherapy (RT) to improve locoregional tumor control is limited by the detrimental effect of higher radiation exposure of thoracic organs-at-risk (OAR). This narrow therapeutic ratio may be expanded by exploiting the advantages of magnetic resonance (MR) linear accelerators, mainly the online adaptation of the treatment plan to the current anatomy based on daily acquired MR images. However, MR-guidance is both labor-intensive and increases treatment times, which raises the question of its clinical feasibility to treat LA-NSCLC. Therefore, the PUMA trial was designed as a prospective, multicenter phase I trial to demonstrate the clinical feasibility of MR-guided online adaptive RT in LA-NSCLC.

**Methods:**

Thirty patients with LA-NSCLC in stage III A-C will be accrued at three German university hospitals to receive MR-guided online adaptive RT at two different MR-linac systems (MRIdian Linac®, View Ray Inc. and Elekta Unity®, Elekta AB) with concurrent chemotherapy. Conventionally fractioned RT with isotoxic dose escalation up to 70 Gy is applied. Online plan adaptation is performed once weekly or in case of major anatomical changes. Patients are followed-up by thoracic CT- and MR-imaging for 24 months after treatment. The primary endpoint is twofold: (1) successfully completed online adapted fractions, (2) on-table time. Main secondary endpoints include adaptation frequency, toxicity, local tumor control, progression-free and overall survival.

**Discussion:**

PUMA aims to demonstrate the clinical feasibility of MR-guided online adaptive RT of LA-NSCLC. If successful, PUMA will be followed by a clinical phase II trial that further investigates the clinical benefits of this approach. Moreover, PUMA is part of a large multidisciplinary project to develop MR-guidance techniques.

**Trial registration:**

ClinicalTrials.gov: NCT05237453.

## Background

Most patients with locally-advanced NSCLC (LA-NSCLC) are ineligible for tumor resection, so that definitive (chemo)radiotherapy (CRT), if possible with consecutive immunotherapy (IT), is the treatment of choice [1]. Despite such multimodal strategies, patients face a dismal prognosis and local tumor relapse remains a major pattern of recurrence [2–5]. To complicate matters, many patients are ineligible for chemotherapy [6], and consecutive IT requires at least stable disease after (C)RT as well as the absence of higher-grade pulmonary toxicity [1]. Therefore, both effectiveness and tolerability of RT essentially determine patient outcomes. RT dose escalation was suggested to be beneficial in LA-NSCLC by several previous reports [7–9], and is also supported by the high local tumor control rates reached after stereotactic ablative RT (SABR) of early-stage NSCLC [10]. However, a recent prospective randomized phase III trial could not demonstrate a clinical benefit of RT dose escalation in LA-NSCLC – on the contrary, overall survival (OS) was even reduced [11]. This surprising result is mainly attributed to a higher dose exposure of thoracic organs-at-risk (OAR) with consecutive treatment-related morbidity [12–14]. Thus, radiation techniques need to be individualized to intensify the dose inside the target volumes while protecting sensitive OAR. Adaptive radiotherapy (ART) accounts for changes in tumor size and intrathoracic anatomy during treatment and allows customization of treatment plans to these changes [15–20]. Thus, ART can protect sensitive OAR [17, 19, 21], which could support dose intensification inside the target [18, 22]. While previous approaches to ART for LA-NSCLC are CT-based and performed offline [17, 20, 21], we propose using online magnetic-resonance (MR)-guidance. Combinations of MR-scanners with linear accelerators, called MR-linacs, allow daily MR-imaging before and during each RT fraction [23, 24]. Hence, treatment plans can be adapted to the anatomy of the day while the patient is lying on the couch (online adaptation) [24–27]. Furthermore, some MR-linac systems already allow gated dose delivery, so that the radiation beam is only activated when the tumor is localized in the correct prespecified position. This may replace internal target volume (ITV) approaches, thus reducing safety margins around the target volume and significantly decreasing dose inside healthy lung tissue [28, 29]. However, MR-guided RT is labor-intensive and may considerably increase treatment times [27, 30], and the successful clinical implementation of MR-guided RT to treat LA-NSCLC has not been shown, yet. Therefore, we designed PUMA (*ClinicalTrials.gov: NCT05237453*) as a prospective, multicenter phase I trial to demonstrate the clinical feasibility of MR-guided online adaptive RT of LA-NSCLC.

## Design

### Objectives and endpoints

The primary objective of the PUMA trial is to demonstrate the clinical feasibility of MR-guided online-adaptive radiotherapy of LA-NSCLC. Clinical feasibility is defined by a two-step approach: (1) successful completion of all but one online adapted fraction in ≥ 80% of patients (≤ 1 cancellations in ≥ 80% of patients), (2) mean treatment duration of online adapted fractions < 90 min. Consequently, the primary endpoints consist of the successful completion as well as the treatment time of each online adapted fraction.

Main secondary endpoints include treatment-related toxicity according to the Common Terminology Criteria for Adverse Events (CTCAE, version 5.0), frequency of major anatomical changes on daily MRI, local tumor control, patterns of tumor recurrence, progression-free survival, overall survival, patient-reported outcomes and pulmonary function. Furthermore, dosimetric comparisons of the MR-guided ART plans with simulated standard-of-care radiotherapy plans (CT-based, non-adaptive RT) as well as with non-adapted MR-guided plans will be performed.

### Patients

30 patients with LA-NSCLC will be equally enrolled at three large German university hospitals (10 patients at each center). Table 1 summarizes all inclusion and exclusion criteria.

**Table 1:** Inclusion and Exclusion Criteria.

Inclusion Criteria	Exclusion Criteria
Histologically-proven NSCLC	Involvement of supraclavicular lymph nodes
Tumor stage III A – C according to the UICC TNM classification (8th edition)	Additional pulmonary lesion (in the same or another lobe)
Indication for definitive thoracic CRT	Previous thoracic RT, if previous and current target volumes overlap
Age ≥ 18 years	Patients who have not yet recovered from acute toxicities of prior therapies
ECOG Score 0–2 (KPI ≥ 70%)	Contraindications against MRI scans
Adequate pulmonary function for CRT	
Ability to lie still on the MR-linac table ≥ 1 h	
Ability to hold one’s breath > 20 s	
Successful completion of MR-guided RT simulation on the treatment machine	

NSCLC: non-small cell lung cancer, UICC: Union for International Cancer Control, (C)RT: (chemo)radiotherapy, ECOG: Eastern Cooperative Oncology Group, KPI: Karnofsky Performance Index, MRI: magnetic resonance imaging

### Chemoradiotherapy

Patients are treated on two different commercially available MR-linac systems: the MRIdian Linac® (0.35 Tesla (T), 6 megavolt (MV) linac; View Ray Inc., Mountain View, CA, USA) and the Elekta Unity® (1.5 T, 7 MV linac; Elekta AB, Stockholm, Sweden). Radiotherapy planning includes a treatment simulation on the respective MR-linac system to assess the patient’s tolerance of MR-guided radiotherapy and to perform a planning MRI in treatment position with arms placed above the head. Successful completion of this treatment simulation is mandatory for trial inclusion. The MRIdian Linac® employs True Fast Imaging with Steady State Precession (TRUFI) sequences, which include a 3D MRI in inspiration breath-hold (resolution: 1.5 × 1.5 mm^2^, slice thickness: 3 mm, breath-hold: 17–25s) as well as 2D cineMRI (resolution: 0.243 × 0.70 cm^2^, 4–8 frames/s) [24]. The Elekta Unity employs a T2-weighted 3-dimensional turbo spin echo (TSE) sequence with compressed sensing in free-breathing (resolution: 2 × 2 × 2.4 mm³). Furthermore, a planning CT-scan (contrast-enhanced, inspiration breath-hold), a diagnostic MRI (3 Tesla, contrast-enhanced, T1-/T2-/diffusion-weighted sequences, inspiration breath hold or free breathing) and a fluorodeoxyglucose positron emission tomography (^18^FDG-PET)-CT scan (< 3 weeks before RT start, acquisition in free breathing, if possible in treatment position) will be performed. Target volume delineation is largely based on the PET-plan trial guidelines [31]. The gross tumor volume (GTV) of the primary (GTV-P) is delineated using all available imaging data, and is expanded by 5 mm while respecting anatomical borders to obtain the clinical target volume (CTV) of the primary (CTV-P). Another CTV is created to encompass all involved lymph node regions (according to the International Association for the Study of Lung Cancer: IASLC [32]) with histologically-proven tumor spread and/or suspicious FDG-uptake (CTV-IN). The CTV-IN is further expanded to include lymph node regions connecting the primary with the obviously involved lymph node regions. Finally, institutional margins are added to obtain the planning target volume (PTV). Main OAR and their dose constraints are defined according to international standards (Table 2).

**Table 2:** Dose Constraints.

Organ at risk	Dose constraints (30–35 fractions)
**Brachial plexus**	D_max_ ≤ 60 Gy
**Esophagus**	D_mean_ ≤ 34 Gy D_max_ ≤ 105% of the prescribed dose
**Heart**	D_mean_ ≤ 20 Gy V_50Gy_ ≤ 25%
**Non-GTV lung**	V_20Gy_ < 35–40% D_mean_ ≤ 20 Gy
**Spinal cord**	D_max_ ≤ 45 Gy

D_max_: maximum dose (appropriate near-maximum dose constraints may be used instead), D_mean_: mean dose, V_20/50Gy_: Relative organ volume receiving > 20/50 Gy

Treatment is performed as conventionally fractionated RT in 2 Gy single doses during weekdays. A total dose of 70 Gy inside the PTV is aimed for, but OAR constraints are prioritized. If OAR constraints cannot be met, the prescribed total dose will be reduced in 2 Gy decrements towards 60 Gy until OAR dose constraints are met (isotoxic dose (de-)escalation). If OAR constraints can still not be met with a prescribed total dose of 60 Gy, PTV coverage will be constrained as much as necessary to comply with the OAR constraints.

At the beginning of each treatment session, a daily MRI is performed in treatment position. Online adaptation is performed once a week or if deemed necessary by the treating physician due to major anatomical changes visible on daily MRI (e.g. new or resolving atelectasis, significant tumor shrinkage). For this purpose, the planning CT-scan is deformably registered to the MRI of the day and the baseline treatment plan is imported. The target volumes are edited on the daily MRI following a “no-shrinking approach” for the CTVs to ensure effective treatment of microscopic disease spread. OAR lying within 3 cm from the PTV (1 cm in craniocaudal direction) will be recontoured according to a PTV_expand_ concept [33]. Finally, the RT plan is adapted using the same planning objectives as the baseline plan with a short online quality assurance (QA) before starting dose application. The treatment team during each session will consist of at least one RTT, one radiation oncologist and one medical physicist. After each online adapted treatment session, adapted plans will additionally undergo offline QA with complete editing of all OAR contours to ensure precise dose quantification inside the OAR (particularly the mean doses of the lung (MLD) and heart (MHD)). Gated dose delivery will be performed on both systems.

Chemotherapy may be administered sequentially or simultaneously, according to institutional standards.

### Follow-up

**Fig. 1:**
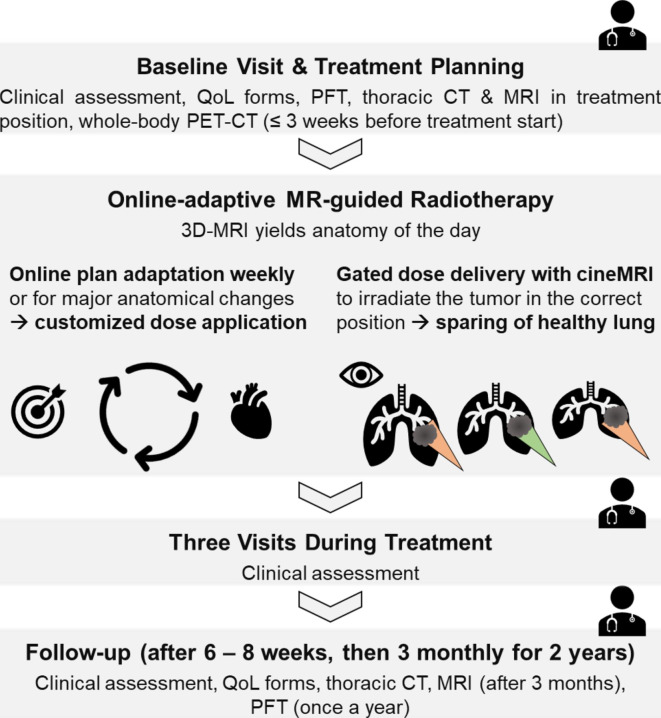
Trial Workflow. QoL: quality-of-life, PFT: pulmonary function testing, CT: computed tomography, MRI: magnetic resonance imaging, PET: positron emission tomography

Patients will be scheduled for clinical visits two and four weeks after treatment start as well as at the last treatment day. Consecutively, patients will be followed-up 6–8 weeks after completion of RT and then 3-monthly for at least 24 months. Follow-up visits encompass clinical examination, quality-of-life questionnaires, thoracic CT as well as pulmonary function tests once a year. Furthermore, another thoracic MRI (1.5 or 3 T, contrast-enhanced, T1-/T2-/diffusion-weighted sequences) will be performed 3 months after treatment completion. Figure 1 summarizes the conduct of the trial.

### Statistics

The first step of clinical feasibility assessment is to demonstrate that all but one online adapted fraction are successfully completed in ≥ 80% of patients (≤ 1 cancellations in ≥ 80% of patients). This proportion of patients will be calculated together with the corresponding 95%-confidence interval using exact Clopper-Pearson boundaries. Secondly, the mean treatment duration should be < 90 min. Thus, the mean averaged on-table times of all patients will be calculated with a corresponding 95%-confidence interval for the mean of a normal distribution. Secondary endpoints will be evaluated with appropriate statistical methods. The interpretation of the results is completely descriptive.

Given the absence of prior data, a formal sample size calculation is not applicable for this phase I feasibility trial. Instead, we estimated the width of the 95%-confidence intervals of the two primary endpoints based on a sample size of 30 patients and assumptions derived from hitherto experience with MR-guided stereotactic radiotherapy to small pulmonary tumors. We obtained reasonable widths to develop future phase II/III trials based on the expected data of the PUMA trial.

## Discussion

The main lesson learned from the RTOG 0617 trial is that if you escalate doses to locally-advanced lung tumors, you also risk elevated doses inside thoracic OAR, which negatively affect patient outcomes. Thus, it seems highly recommendable to increase the conformality of thoracic RT, especially when attempting to intensify the dose inside the target volumes [11, 13]. Several previous studies have shown the potential of ART to customize dose application to LA-NSCLC with favorable clinical outcomes [17–22], and one recent study even suggested a survival benefit compared to non-adaptive treatment [34]. However, a prospective comparison of the clinical outcomes between ART and non-adaptive RT in LA-NSCLC has not been completed, yet.

While almost all previously published experiences of ART for LA-NSCLC are cone-beam CT (CBCT) or CT-based [17, 19–21], data on MR-guided ART is scarce. But the use of MR-guidance instead of CT-guidance offers crucial advantages. Firstly, it is well-known that MRI allows for superior soft tissue contrast, which is essential for precise localization of mediastinal organs such as the esophagus or heart. Moreover, multiparametric MRI serves as useful staging method in NSCLC and may be a potential alternative to PET-CT according to the Fleischner Society [35]. Correspondingly, several previous works suggest that MR-guided online adaptation can effectively protect sensitive thoracic OAR close to smaller lung tumors treated with SABR [25, 27, 36]. MRI also works without ionizing radiation, which minimizes the exposure of the whole body to low radiation doses. Furthermore, this enables cineMRI during dose delivery, so that the correct position and trajectory of the tumor can be verified during treatment. The MRIdian Linac® system already allows for gating of the treatment beam, which obviated the need for ITV concepts and thus significantly reduces doses to healthy lung tissue during SABR [28, 29]. Such a reduction of healthy lung doses has shown to reduce pulmonary toxicity for similar respiratory gating techniques [37]. However, MR-guidance also faces technical constraints, such as motion artifacts, as well as major clinical limitations. Treatment times increase considerably due to online plan adaptation and potentially gated dose delivery, which reduces patient comfort. In addition, gated dose delivery requires a focused, reproducible breathing pattern with repeated breath holds, which limits its application to patients with adequate pulmonary function and good compliance with breathing commands. Therefore, the feasibility of MR-guided ART to treat LA-NSCLC is not clear yet. Previous experiences with the successful and beneficial use of pulmonary SABR [26, 29], including patients with constrained pulmonary function [27], led us to design the PUMA trial and to define clinical feasibility in a two-step approach including the successfully applied online adapted fractions and the required time frames for online adaptation.

Currently, another clinical trial investigates an adaptive MR-guided approach towards hypofractionated CRT in patients with LA-NSCLC (*ClinicalTrials.gov: NCT03916419*). Hypofractionation leads to shortened overall treatment times and could thus improve local tumor control from a radiobiological perspective [38–41]. However, several phase I/II clinical trials have suggested higher rates of severe toxicity compared to conventionally fractionated CRT [39–41]. Again, it seems that the dose exposure of thoracic OAR is the limiting factor. Therefore, the combination of hypofractionated RT with MR-guidance might be a promising liaison. MR-guidance yields the precision to tailor dose distribution to the current anatomy, which protects sensitive OAR and widens the therapeutic ratio. At the same time, hypofractionated schedules significantly reduce the number of fractions and thus overall treatment times, which compensates for the increased duration of a single fraction. The PUMA trial aims to demonstrate the clinical feasibility of MR-guided online adaptive RT as a first step. Hence, we have chosen a conventionally fractionated RT approach with an isotoxic dose intensification within the range of our national S3 guideline [42]. Isotoxic dose escalations have proven feasible without excessive toxicity in previous trials [31, 43].

The optimum frequency of plan adaptation in ART of NSCLC is still unclear [17, 21, 44]. In the LARTIA trial, 23% of patients underwent re-planning due to CBCT-based tumor shrinkage after a median dose of 45 Gy equivalent dose in 2 Gy fractions (EQD_2_), but with a wide dose range (20–60 Gy EQD_2_) [21]. Møller et al. reported at least one plan adaptation in 33% of their patients due to anatomical and/or tumor changes on CBCT at different time points, with most adaptations after 6–20 fractions [17]. However, plan adaptation may be indicated both earlier and more often when daily thoracic MRI with higher soft tissue contrast becomes available. We have chosen to perform plan adaptation once weekly or in case of major anatomical changes on daily MRI. Moreover, we have decided to implement an online adaptation approach, which considerably increases the duration of a treatment fraction. To avoid excessively long treatment fractions, we will follow a PTV_expand_ concept [33], but this also introduces uncertainties regarding the mean doses inside the lungs and heart (MLD, MHD). Therefore, additional offline QA will be performed to verify the online adapted plans. However, online plan adaptation immediately and precisely customizes the dose distribution to the daily anatomy. This supports approaches towards dose escalation and particularly hypofractionation, which can in turn compensate for the increased treatment times per fraction. In summary, the design of the PUMA trial allows to define a reasonable future adaptation strategy, which includes the exploration of (1) optimum time points for plan adaptation, (2) major anatomical changes and their association with clinically relevant dose deviations, (3) the most-reasonable implementation of plan adaptation (online versus offline), (4) dosimetric advantages compared to a non-adaptive CT and MR-based approaches.

Whether target volumes and thus dose coverage can be safely adapted to a shrinking tumor remains a matter of debate. Results from the LARTIA trial suggest a low rate of marginal failures when adapting target volumes to the shrinking tumor [21], which is in line with a previous planning study [22]. Nevertheless, we decided to implement a “no-shrinking-approach” for the CTVs to ensure safe dose coverage of microscopic disease in this early clinical trial.

The limitations of MR-guidance have mostly been discussed above and are reflected by the inclusion and exclusion criteria. Long treatment times and good patient compliance concerning breathing commands require successful completion of an initial treatment simulation. Patients with implants incompatible with magnetic field are ineligible for MR-guided RT. Moreover, some MR-linac systems offer limited fields-of-view and RT field sizes, so that LA-NSCLC with involvement of supraclavicular lymph nodes or pulmonary satellite lesions need to be excluded as well. Another limitation is the use of different MR-linac systems with application of different institutional PTV margins, which might bias target volume size and thus clinical endpoints. Many of these technical limitations may be improved in the future, e.g. by manufacturing MR-linacs with larger field sizes or by reducing treatment times through AI-based automation of online contouring and plan adaptation. Therefore, the PUMA trial lies at the heart of a large multidisciplinary project that aims to develop MR-guided RT techniques. Moreover, we plan to design a clinical phase II trial based on the clinical feasibility and outcome data of PUMA to further investigate the benefits of MR-guided RT for treatment-related toxicity and local tumor control.

## Data Availability

Not applicable.

## Notes

The original version of this article was revised: that the article title was incomplete in the original publication.
