# Correction : Pulmonary magnetic resonance-guided online adaptive radiotherapy of locally advanced non-small cell lung cancer: the PUMA trial

**DOI:** 10.1186/s13014-023-02294-5

**Published:** 2023-06-08

**Authors:** Sebastian Regnery, Chiara de Colle, Chukwuka Eze, Stefanie Corradini, Christian Thieke, Oliver Sedlaczek, Heinz-Peter Schlemmer, Julien Dinkel, Ferdinand Seith, Annette Kopp-Schneider, Clarissa Gillmann, C. Katharina Renkamp, Guillaume Landry, Daniela Thorwarth, Daniel Zips, Claus Belka, Oliver Jäkel, Jürgen Debus, Juliane Hörner-Rieber

**Affiliations:** 1 Department of Radiation Oncology, Heidelberg University Hospital, Im Neuenheimer Feld 400, 69120 Heidelberg, Germany; 2 National Center for Radiation Oncology (NCRO), Heidelberg Institute for Radiation Oncology (HIRO), Im Neuenheimer Feld 400, 69120 Heidelberg, Germany; 3 Department of Radiation Oncology, Heidelberg Ion-Beam Therapy Center (HIT), Heidelberg University Hospital, Heidelberg, Germany; 4 National Center for Tumor diseases (NCT), Heidelberg, Germany; 5 Clinical Cooperation Unit Radiation Oncology, German Cancer Research Center (DKFZ), Heidelberg, Germany; 6 Department of Radiation Oncology, University Hospital Tübingen, Tübingen, Germany; 7 Department of Radiation Oncology, University Hospital, LMU Munich, Munich, Germany; 8 Division of Radiology, German Cancer Research Center (DKFZ), Heidelberg, Germany; 9 Department of Radiology, LMU Munich, Munich, Germany; 10 Department of Radiology, University Hospital Tübingen, Tübingen, Germany; 11 Division of Biostatistics, German Cancer Research Center (DKFZ), Heidelberg, Germany; 12 Division of Medical Physics in Radiation Oncology, German Cancer Research Center (DKFZ), Heidelberg, Germany; 13 Section for Biomedical Physics, Department of Radiation Oncology, University Hospital Tübingen, Tübingen, Germany

**Correction: Radiat Oncol 18, 74 (2023)** 10.1186/s13014-023-02258-9

Following publication of the original article [1], it was reported that the article title was incomplete. The correct title is given in this Correction and the original article has been updated.
